# Supplementary material for: The Systems Biology Research Tool: evolvable open-source software
Source: BMC Syst Biol. 2008 Jun 29;2:55. doi: 10.1186/1752-0509-2-55 (PMC2446383; doi:10.1186/1752-0509-2-55)
Supplement: Additional file 1 — SBRT Archive. An archive of the current version of the Systems Biology Research Tool. [file 1752-0509-2-55-S1.zip › sbrt-1.4.0/doc/developers_guide/api/sbrt/kernel/math/algebra/MatrixWrap.html]

MatrixWrap


|  |  |  |
| --- | --- | --- |
| **Overview** \| **Package** \| **Class** \| **Use** \| **Tree** \| **Deprecated** \| **Index** \| **Help** | |  |
| **PREV CLASS**   **NEXT CLASS** | **FRAMES**    **NO FRAMES**     **All Classes** |  |
| SUMMARY: NESTED \| FIELD \| CONSTR \| METHOD | DETAIL: FIELD \| CONSTR \| METHOD |  |


---


## sbrt.kernel.math.algebra Class MatrixWrap<R,C,E>

```
java.lang.Object
  sbrt.kernel.math.algebra.MatrixWrap<R,C,E>
```

**Type Parameters:**: `R` - the row type.: `C` - the column type.: `E` - the element type.

**All Implemented Interfaces:**: Matrix<R,C,E>

**Direct Known Subclasses:**: DoubleMatrixWrap

---

``` public class MatrixWrap<R,C,E> extends java.lang.Object implements Matrix<R,C,E> ```

This class is used to wrap mutable matrices as an immutable
matrix. This type of wrapping eliminates the possibility of
casting a mutable matrix back to its mutable type. This ensures that
a mutable matrix can be viewed by others in a safe way, without
fear of modification.

Instances of this class are immutable.

**Author:**
:   This class was written and documented by
    Jeremiah Wright while in the Wagner lab.

---

**Constructor Summary**

`MatrixWrap(Matrix<R,C,E> m)`
  
          Constructs a new wrapper around the provided matrix.


| **Method Summary** | |
| --- | --- |
| `int` | `columns()`             Returns the number of columns in this matrix. |
| `boolean` | `equals(java.lang.Object obj)`             Indicates if the provided object is equal to this matrix. |
| `Matrix<R,C,E>` | `getColumn(java.lang.Object columnIndex)`             Returns the column corresponding to the provided column index. |
| `UnmodifiableSet<C>` | `getColumnIndices()`             Returns the set of column indices contained in this matrix. |
| `E` | `getElement(java.lang.Object rowIndex, java.lang.Object columnIndex)`             Returns the element of this matrix at the provided row and column indices. |
| `Matrix<R,C,E>` | `getRow(java.lang.Object rowIndex)`             Returns the row corresponding to the provided row index. |
| `UnmodifiableSet<R>` | `getRowIndices()`             Returns the set of row indices contained in this matrix. |
| `int` | `hashCode()`             Returns a content-based hash code for this matrix. |
| `boolean` | `isColumnIndex(java.lang.Object obj)`             Indicates if the provided object is a column index in this matrix. |
| `boolean` | `isEmpty()`             Indicates if this matrix is empty. |
| `boolean` | `isRowIndex(java.lang.Object obj)`             Indicates if the provided object is a row index in this matrix. |
| `int` | `rows()`             Returns the number of rows in this matrix. |
| `java.lang.String` | `toString()`             Returns a string representation of this matrix. |

**Methods inherited from class java.lang.Object**

`clone, finalize, getClass, notify, notifyAll, wait, wait, wait`

**Constructor Detail**

### MatrixWrap

```
public MatrixWrap(Matrix<R,C,E> m)
```

:   Constructs a new wrapper around the provided matrix.

    **Parameters:**: `m` - the matrix to be wrapped by this wrapper object. **Throws:**: `java.lang.NullPointerException` - if the provided matrix is `null`.


**Method Detail**

### getElement

```
public E getElement(java.lang.Object rowIndex,
                    java.lang.Object columnIndex)
```

:   Returns the element of this matrix at the provided
    row and column indices.

    :   **Specified by:**: `getElement` in interface `Matrix<R,C,E>`
    :   **Parameters:**: `rowIndex` - the row index.: `columnIndex` - the column index. **Returns:**: the element of this matrix at the provided row and column indices.

---


### getColumnIndices

```
public UnmodifiableSet<C> getColumnIndices()
```

:   Returns the set of column indices contained in this
    matrix.

    :   **Specified by:**: `getColumnIndices` in interface `Matrix<R,C,E>`
    :   **Returns:**: the set of column indices contained in this matrix.

---


### getRowIndices

```
public UnmodifiableSet<R> getRowIndices()
```

:   Returns the set of row indices contained in this
    matrix.

    :   **Specified by:**: `getRowIndices` in interface `Matrix<R,C,E>`
    :   **Returns:**: the set of row indices contained in this matrix.

---


### isRowIndex

```
public boolean isRowIndex(java.lang.Object obj)
```

:   Indicates if the provided object is a row index in
    this matrix.

    :   **Specified by:**: `isRowIndex` in interface `Matrix<R,C,E>`
    :   **Parameters:**: `obj` - the potential row index. **Returns:**: `true` if the provided object is a row index in this matrix; `false` otherwise.

---


### isColumnIndex

```
public boolean isColumnIndex(java.lang.Object obj)
```

:   Indicates if the provided object is a column index in
    this matrix.

    :   **Specified by:**: `isColumnIndex` in interface `Matrix<R,C,E>`
    :   **Parameters:**: `obj` - the potential column index. **Returns:**: `true` if the provided object is a column index in this matrix; `false` otherwise.

---


### getRow

```
public Matrix<R,C,E> getRow(java.lang.Object rowIndex)
```

:   Returns the row corresponding to the provided row index.
    A row is itself a 1 x n matrix, where n is the number of
    columns in this matrix.

    :   **Specified by:**: `getRow` in interface `Matrix<R,C,E>`
    :   **Parameters:**: `rowIndex` - the index of the row to be returned. **Returns:**: the row corresponding to the provided row index.

---


### getColumn

```
public Matrix<R,C,E> getColumn(java.lang.Object columnIndex)
```

:   Returns the column corresponding to the provided column index.
    A column is itself an m x 1 matrix, where m is the number of
    rows in this matrix.

    :   **Specified by:**: `getColumn` in interface `Matrix<R,C,E>`
    :   **Parameters:**: `columnIndex` - the index of the column to be returned. **Returns:**: the column corresponding to the provided column index.

---


### rows

```
public int rows()
```

:   Returns the number of rows in this matrix.

    :   **Specified by:**: `rows` in interface `Matrix<R,C,E>`
    :   **Returns:**: the number of rows in this matrix.

---


### columns

```
public int columns()
```

:   Returns the number of columns in this matrix.

    :   **Specified by:**: `columns` in interface `Matrix<R,C,E>`
    :   **Returns:**: the number of columns in this matrix.

---


### isEmpty

```
public boolean isEmpty()
```

:   Indicates if this matrix is empty.

    :   **Specified by:**: `isEmpty` in interface `Matrix<R,C,E>`
    :   **Returns:**: `true` if at least one row and column exists in this matrix; `false` otherwise.

---


### toString

```
public java.lang.String toString()
```

:   Returns a string representation of this matrix.

    :   **Specified by:**: `toString` in interface `Matrix<R,C,E>` **Overrides:**: `toString` in class `java.lang.Object`
    :   **Returns:**: a string representation of this matrix.

---


### equals

```
public boolean equals(java.lang.Object obj)
```

:   Indicates if the provided object is equal to this matrix.
    Two matrices are considered equal if they share the same
    sets of column and row indices and the elements in each
    matrix for each column-row index pair are the same.

    :   **Specified by:**: `equals` in interface `Matrix<R,C,E>` **Overrides:**: `equals` in class `java.lang.Object`
    :   **Parameters:**: `obj` - the object with which to compare. **Returns:**: `true` if this object equals the provided object; `false` otherwise.

---


### hashCode

```
public int hashCode()
```

:   Returns a content-based hash code for this matrix.

    :   **Specified by:**: `hashCode` in interface `Matrix<R,C,E>` **Overrides:**: `hashCode` in class `java.lang.Object`
    :   **Returns:**: a content-based hash code for this matrix.


---


|  |  |  |
| --- | --- | --- |
| **Overview** \| **Package** \| **Class** \| **Use** \| **Tree** \| **Deprecated** \| **Index** \| **Help** | |  |
| **PREV CLASS**   **NEXT CLASS** | **FRAMES**    **NO FRAMES**     **All Classes** |  |
| SUMMARY: NESTED \| FIELD \| CONSTR \| METHOD | DETAIL: FIELD \| CONSTR \| METHOD |  |


---
